# The semantic structure of accuracy in eyewitness testimony

**DOI:** 10.3389/fpsyg.2024.1211987

**Published:** 2024-04-10

**Authors:** Philip U. Gustafsson, Sverker Sikström, Torun Lindholm

**Affiliations:** ^1^Department of Psychology, Stockholm University, Stockholm, Sweden; ^2^Department of Psychology, Lund University, Lund, Sweden

**Keywords:** eyewitness testimony, eyewitness accuracy, semantic content, LSA, BERT

## Abstract

In two studies, we examined if correct and incorrect statements in eyewitness testimony differed in semantic content. Testimony statements were obtained from participants who watched staged crime films and were interviewed as eyewitnesses. We analyzed the latent semantic representations of these statements using LSA and BERT. Study 1 showed that the semantic space of correct statements differed from incorrect statements; correct statements were more closely related to a *dominance* semantic representation, whereas incorrect statements were more closely related to a *communion* semantic representation. Study 2 only partially replicated these findings, but a mega-analysis of the two datasets showed different semantic representations for correct and incorrect statements, with incorrect statements more closely related to representations of *communion* and *abstractness.* Given the critical role of eyewitness testimony in the legal context, and the generally low ability of fact-finders to estimate the accuracy of witness statements, our results strongly call for further research on semantic content in correct and incorrect testimony statements.

## 1 Introduction

Language enables us to share information and recount events that we have experienced. This ability is crucial in courtrooms, where eyewitnesses commonly testify to what they have seen during criminal events. However, witnesses can lie or misremember, which makes it important to evaluate the accuracy of a testimony. Given the centrality of language in communication, it is not surprising that researchers have attempted to find verbal cues to identify if a witness is lying (see DePaulo et al., 2003), or to tie a suspect to a threat letter (Nini, 2018). In this study, we analyze the verbal content of testimonies from eyewitnesses and examine how correct and incorrect statements might differ semantically.

### 1.1 Predicting eyewitness accuracy with semantic content

Although much research on accuracy in eyewitness testimonies concerns deception detection (for meta-analyses, see DePaulo et al., 2003; Sporer and Schwandt, 2006; Vrij et al., 2017), another important area of research is predicting accuracy in eyewitnesses, as people can remember incorrectly. A common method to evaluate eyewitness accuracy is to ask witnesses to describe how certain they are in the accuracy of their memory, that is, make a confidence judgment. Overall, much evidence suggest that confidence can be a good indicator of accuracy, such that high confidence is given to correct answers (e.g., Juslin et al., 1996; Mickes et al., 2017; Wixted and Wells, 2017; but see also Wade et al., 2018). There are however also other ways to evaluate eyewitness accuracy, such as by directly analyzing the content of the testimony itself. In theory, methods utilizing such an approach should have the possibility to generate more accurate predictions than confidence judgments, as a testimony is a more direct output of memory, and should therefore contain less error. That is, confidence is a metacognitive judgment and as such, is based not just on appraisals of the memory, but also on knowledge and beliefs (e.g., Flavell, 1979; Mueller et al., 2013). In contrast, a testimony is a direct verbal report of memory contents, and is therefore closer to the source (i.e., a correct or incorrect memory). So far, studies analyzing testimony content have evaluated the testimony’s *semantic* content. Here follows a summary of two such methods.

The first method to predict accuracy in eyewitness testimony from semantic content is based on the reality-monitoring framework (Johnson and Raye, 1981). With this method, researchers analyze the types of details that are expressed in the testimony. Specifically, the idea is that a real (correct) memory will contain more sensory, spatial and temporal details compared to an imagined (incorrect) memory, which will instead contain more references to cognitive operations (e.g., how one came to remember the detail). For example, participants in an experiment by Schooler et al. (1986) watched a presentation that involved a car at an intersection. Half of the participants saw a yield sign at the intersection, whereas the other half of the participants only had the sign suggested to them in a later questioning. When the participants later had to describe objects in the presentation, those who had seen the yield sign (i.e., gave a correct recall) used twice as many sensory details to describe it, whereas those who only had the sign suggested to them (i.e., gave an incorrect recall) used 10 times as many indications of cognitive operations. Other similar findings support the idea that sensory details are more abundant in correct memories and that references to cognitive operations are more abundant in incorrect memories (Hashtroudi et al., 1990; Stromwall and Granhag, 2005; Kensinger and Schacter, 2006; Sporer and Sharman, 2006; c.f. Clark-Foos et al., 2015). However, a drawback is that this method requires an assessment of the overall testimony credibility, and therefore cannot be used to evaluate the accuracy of individual statements within the testimony.

A more recent method predicts accuracy in eyewitness testimonies based on the amount of expressed effort in retrieving a memory. Early on, Smith and Clark (1993) evaluated answers to general knowledge questions and found that incorrect answers more often contained filler expressions such as “uh,” “oh,” and hedges such as “I do not know,” “I think,” compared to correct answers. Later on, studies examining effort in eyewitness testimonies replicated these findings (Paulo et al., 2015, 2019; Lindholm et al., 2018; Gustafsson et al., 2019, 2022; see also Seale-Carlisle et al., 2022). Moreover, these studies corroborate findings that correct memories are retrieved quicker—that is, easier—than incorrect memories (Robinson et al., 1997; Brewer et al., 2006; Koriat and Ackerman, 2010; Ackerman and Koriat, 2011).

Despite the relative success of the reality-monitoring method and the retrieval-effort method, they are both limited by requiring manual coding; coders must manually process each statement and evaluate the respective semantic cues, which can result in mistakes such as missed cues or incorrect coding. Although this shortcoming is largely mitigated by using coder protocols and interrater reliability tests, it remains a costly and lengthy operation. A possible remedy would therefore be to run eyewitness testimonies through a computerized semantic text analysis.

### 1.2 Data-driven analyses of semantic content

Data-driven text analyses are common in psycholinguistic research. In the field of authorship attribution—that is, the evaluation of whether two or more texts belong to the same author—a central idea is that people have a “written fingerprint” that can be detected by a person’s writing style (i.e., stylometry, Coulthard et al., 2016). To find this “written fingerprint,” researchers analyze the occurrence of various features of a text, such as lexical, syntactic, or semantic features, or some combination of them (see Stamatatos, 2009 for an overview). This is mainly carried out with machine-learning methods (e.g., Zheng et al., 2006; Zlatkova et al., 2018). One such method is based on identifying the frequency of co-occurring letters or words, called n-grams (e.g., Kešelj et al., 2003; Koppel et al., 2009; Johnson and Wright, 2014; Kestemont et al., 2018; Nini, 2018). N-grams are sequential items found in a verbal output. For example, the sentence “He was wearing a blue jacket” with a word 3-gram would result in four sequences: “He was wearing,” “was wearing a,” “wearing a blue, “a blue jacket.” By calculating n-grams over entire texts, you get a library of words (or letters) that commonly occur together. This library can then be compared with libraries constructed from the works of potential authors to examine the overlap. For example, Nini (2018) investigated the authorship of letters that were sent in relation the infamous Whitechapel murders in nineteenth century England. Nini (2018) examined over two hundred letters supposedly sent by “Jack the Ripper” and used word 2-grams to find that the two most famous letters likely came from the same person. Besides examining overlaps, n-grams can also be used—as is the case for the current study—to examine differences between texts.

Initial support for a word gram approach to differentiate correct and incorrect eyewitness statements comes from Sarwar et al. (2015), who utilized word 5-grams together with a method called latent semantic analysis (LSA, Landauer, 2007). The LSA analyses a text and creates a “semantic space” that describes the relation between the words in that text. Words that occur more frequently together in a text (e.g., “cute” and “kitten”) are said to be more closely related in this semantic space, compared to words that occur less frequently together (e.g., “cute” and “capillary”). With this method, Sarwar et al. (2015) examined if words used to describe correct statements belonged in a different semantic space compared to words used to describe incorrect statements. They found this to be the case, thus strengthening the idea that we linguistically express ourselves differently when we remember correctly compared to incorrectly.

Despite the positive results, the study by Sarwar et al. (2015) was limited in two important ways. First, the participants wrote down their memories instead of verbally recalling them. This constrains the ecological validity, as eyewitnesses tend to recall experienced events verbally. Importantly, verbal recall likely contains more genuine, unfiltered expressions than written recall, which could allow for greater semantic differences between correct and incorrect statements. Second, the LSA analysis that showed a difference in semantic space between correct and incorrect statements provided no clue as to what made up this difference. That is, it is unknown if the semantic difference between correct and incorrect statements related to the use of function verbs, expressed emotions, or some other semantic context. Sarwar et al. (2015) commendably attenuated this shortcoming by presenting a list of the most frequent words in incorrect statements. Nonetheless, it is difficult to interpret overarching trends. On one hand, certain words could be categorized as hedges (e.g., “could,” “possibly”), which corroborates previous results (Smith and Clark, 1993; Lindholm et al., 2018; Gustafsson et al., 2019, 2022). On the other hand, many words were general nouns that likely refer to specific details in the mock crime video (e.g. “jacket,” “skirt”) and therefore probably do not generalize well.

In the current study, we aim to replicate the results in Sarwar et al. (2015) by examining the accuracy of statements in eyewitness testimonies with a data-driven method focused on linguistic content, and aim to improve on previous limitations by examining transcripts of eyewitness interviews instead of written eyewitness statements. Furthermore, we will examine specific semantic representations to try to decipher the content of the previously found semantic difference between correct and incorrect statements. Finally, we also analyze data both with LSA, and another natural-language processing technique (“Bidirectional Encoder Representations from Transformers model” [BERT] Devlin et al., 2018) to evaluate the reliability of findings.

## 2 Study 1

The aim of the first study was to investigate the semantic space of correct and incorrect eyewitness statements (see Sarwar et al., 2015). We hypothesized (1) that correct and incorrect statements would occupy different semantic spaces, that is, differ linguistically. Furthermore, we examined the potential content of such a linguistic difference. Our idea was that witnesses might express themselves in a friendlier, warmer style when remembering incorrectly, in order to compensate for a potential feeling of “lesser competence” from providing an incorrect answer. This idea is based on the stereotype content model (Cuddy et al., 2008; see also Abele and Wojciszke, 2007), which suggests that people often evaluate others across two dimensions: communion/warmth (e.g., friendly, kind) and agency/competence (e.g., skillful, intelligent). When people feel that they are underperforming in one dimension, they tend to compensate by emphasizing the other dimension (Holoien and Fiske, 2013; Lindholm and Yzerbyt, 2018). For example, a person that feels low in competence can compensate by appearing friendlier. We expected that this might come into play in an eyewitness context; when witnesses recollect something inaccurately, a feeling of lacking competence should arise. The witness may then express themselves in a “warmer” fashion, and use a more positive tone. We therefore hypothesized (2) that incorrect statements would be more semantically similar to the concept of communion and (3) have a higher valence compared to correct statements. Conversely, recalling correct memories should instill a sense of competence. Witnesses may then express themselves in a more dominant fashion. We therefore also hypothesized (4) that correct statements would be more closely related to a dominant-semantic representation.

### 2.1 Data availability

The data and code for both studies (including the mega analysis) are available at https://osf.io/f83wt/?view_only=5b1ea8cf6d944a7da44fb3f9eb82dc29 and https://osf.io/ztcs8/?view_only=a578b381301541ab9a96b5e444a0eef5.

### 2.2 Method

#### 2.2.1 Dataset

The dataset was originally published in Lindholm et al. (2018) as Study 1, in which 34 participants (*M*_age_ = 31.06; *SD* = 7.37, 100% men) were interviewed in Swedish as witnesses after having watched a mock crime video of a kidnapping. These interviews were videotaped and then transcribed verbatim. The interviews included a free recall phase, immediately followed by cued recall questions (e.g., “how old was the first offender?”). Objectively verifiable statements from answers to the cued recall questions were cataloged and coded for accuracy. The dataset comprised 783 statements (78.16% correct).

The study was conducted in full in accordance with the ethical principles outlined in ALLEA (All European Academies) (2017), and with the ethical principles outlined on http://www.codex.vr.se/, and with the 1964 Helsinki declaration and its later amendments. The studies did not include factors that require ethical vetting according to Swedish legislation on research ethics Svensk författningssamling (2003). All participants had given written informed consent to participate. For a full description of the procedure, see Lindholm et al. (2018).

#### 2.2.2 Data analysis

The cataloged statements were quantified by using a version of the LSA (Landauer, 2007) algorithm as described in Kjell et al. (2019). The semantic space was created from the Swedish version of Google N-gram that is publicly available for download at https://catalog.ldc.upenn.edu/LDC2009T25. In this database we used 5-word grams that are available without further pre-processing. First, a co-occurrence matrix was created, where the columns were the 120,000 most common words, and the rows the 50,000 most common words. Each cell represents the number of times the word in the column and the word in row co-occurs in the 5-word grams. The content of the cells was normalized by taking the logarithm plus one. A data compression algorithm called singular value decomposition (SVD) was then applied to the co-occurrence matrix, with the purpose to maintain as much information in the matrix as possible, in a new matrix that is smaller than the original matrix. This resulted in a semantic space where each of the selected words was described by a vector consisting of 300 dimensions. The length of this vector was normalized to one which is a necessary step to scale the semantic similarity scores between –1 and +1. A semantic representation was created for each eyewitness statement by summing semantic representation in each dimension over all words in the statement. The resulting vector was again normalized to the length of one, by calculating the length of the vector and dividing each dimension with that value. This resulted in a 300-dimensional semantic representation for each statement, each with a length of one.

In addition to creating a semantic space with LSA, statements were also quantified using the Bidirectional Encoder Representations from Transformers model (BERT, Devlin et al., 2018). BERT is a deep neural network language model that unlike LSA generates embedding that handles the grammatical structure of the texts. Thus, BERT acts as a similar, yet more refined method to create semantic spaces. Here we selected the multilingual model “bert-base_multilingual-cased” from Huggingface^1^, and extracted the representation on the last layer (i.e., layer 12) that consisted of 768 dimensions. The length of this vector was normalized to one.

A prediction model for accuracy was created following the method that is specified in Kjell et al. (2019). We first preprocessed the semantic space by applying the SVD algorithm on the vectors representing the eyewitness statements. This was done separately both for the semantic representations generated by BERT and LSA (to maintain a consistent processing of the two different models). We then trained a model to predict accuracy using multiple linear regression, where we optimized the number of dimensions by trying the first; 1, 2, 3, 5, 7, 10, 14, 19, 26, 35, 46, 61, 80, 105, 137, 179, 234, 305, 397, 488, 768 number of dimensions for the BERT model, and up to 300 for the LSA model. The model was then evaluated by using an 11-fold nested cross-validation procedure, where train and test data always was always separated and where optimization of the hyperparameter number-of-dimensions were conducted in the training dataset (i.e., each fold could have different values) set and then applied on the test dataset. The mean number of used dimensions over the folds were 9.5 (with a standard deviation of 4.2) for the BERT and 9.6 for LSA representation (with a standard deviation of 1.5).

To avoid training on the similar information as tested, the folds were selected by manually classifying each fold into one of 11 different themes based on the statements given in the testimonies (e.g., “clothes,” “weapons”). The themes differed in the mean and standard value of correct classification, which could potentially introduce biases in the machine learning algorithm. For example, it could potentially learn differences in the mean values between themes, rather than learn whether individual statements are correct or not. To avoid this potential problem that otherwise would create artifacts in the predictions, we z-transformed the binary accuracy measures (0 and 1) for fold separately. As the mean and the standard deviation is different for each theme, this z-transformation remaps the binary outcome variable to a normal distribution with a mean of zero and standard deviation of one. Thus, following this z-transformation the machine learning problem became a regression model, and not a binary classification problem.

To allow for comparisons between accuracy and semantic measures of communion, valence and dominance, we created semantic representations for each of these constructs. The *communion* representation was created using the English “communion” word list from Pietraszkiewicz et al. (2019). This list contains words such as “emotional,” “feelings,” “modest,” but also “religious” and “tempting.” We then created a semantic representation of this list using the same method as for the semantic representation of the statements, with the exception that we use an English LSA representation with 512 dimensions from Kjell et al. (2019). That is, we summed the semantic representation in each dimension for all words in the list and normalized the length of the resulting vector to one by dividing each dimension with length of the vector. This resulted in a 512-dimensional semantic representation of the list with a length of one. Following the recommendation of Boyd et al. (2022), we used Google to translate the original data from the participants from Swedish to English.

For *valence* and *dominance* we used a list of words in Swedish that were rated on these constructs, as such word lists were available. The *dominance* measure (ranging from 1 to 9) was created from a Swedish wordlist (Waldhauser, 2022), where participants rated words (*N* = 857) for their degree of dominance. Examples of high rated words are “alliance,” “speaker,” and “sadist,” and examples of low rated words are “rabbit,” “belief,” and “toy.” The same predictive model as specified above, with the exception that no *z*-transformation was conducted (i.e., multiple linear regression, see also Kjell et al., 2019) was trained to predict the ratings from the semantic representation of the words. The ten-percentage leave-out cross-validation procedure generated a significant correlation between predicted and rated dominance, *r* = 0.27, *p* < 0.001. This model was applied to the text in the current dataset to predict the dominance measure. The *valence* measure (ranging from-3 to +3) was created using the same method (*r* = 0.67, *p* < 0.001), based on a Swedish word list collected by (Stenberg, 2006, *N* = 288). Examples of words with high (positive) valence are “wisdom,” “love,” and “trust,” and examples of words with low valence are “cancer,” “torture,” and “murder.” The prediction accuracy of the dominance ratings is lower than for valence, which may be because dominance ratings are more difficult to conduct compared to valence ratings. This idea is supported by the variability of dominance ratings, which, averaged over subjects, are lower than for the valence ratings.

### 2.3 Results and discussion

#### 2.3.1 Accuracy and semantic spaces

We t-tested whether the predicted accuracy differed between correct and incorrect statements (Hypothesis 1). This was done by conducting a t-test comparing the predicted accuracy between correct and incorrect statements in the BERT and LSA-models. The results were statistically significant both with the BERT analysis, *t*(846) = −4.65, *p* < 0.001, *d* = 0.39, *MSE* = 0.88 (see Table 1), and with the LSA analysis, *t*(846) = −4.35, *p* < 0.001, *d* = 0.37, *MSE* = 0.90 (see Table 1), indicating that correct and incorrect statements differ in semantic content.

**Table 1:** Semantic measures of accuracy.

Study	Measure	Language	*N*	*I*	*C*	*p*	*t*	Cohen’s d [-CI, +CI]	MSE
1	Accuracy (LSA)	swe	847	−0.05	0.04	0.0000**	−4.35	−0.37 [−0.12, −0.05]	0.90
1	Accuracy (BERT)	swe	847	−0.04	0.05	0.0000**	−4.65	−0.39 [−0.13, −0.05]	0.88
1	Valence	swe	837	6.13	6.09	0.4922	0.69	0.06 [−0.07, −14]	
1	Dominance	swe	837	0.03	0.04	0.0003**	−3.66	−0.31 [−0.02, −0.01]	
1	Abstract	swe	837	0.14	0.13	0.1568	1.42	0.12 [0.00, 0.03]	
1	Communion	eng	844	0.23	0.20	0.0016*	3.16	0.27 [0.01, 0.04]	
1	Tentativeness	eng	844	−0.02	−0.02	0.7262	0.35	0.03 [−0.01, 0.01]	
2	Accuracy (LSA)	swe	1,535	−0.11	0.01	0.0000**	−8.29	−0.48 [−0.15, −0.09]	1.06
2	Accuracy (BERT)	swe	1,535	−0.05	−0.01	0.0004**	−3.52	−0.21 [−0.07, 0.02]	1.07
2	Valence	swe	1,530	6.05	6.10	0.2145	−1.24	−0.07 [−0.12, −03]	
2	Dominance	swe	1,530	0.03	0.03	0.0519	1.95	0.12 [0.00, 0.01]	
2	Abstract	swe	1,530	0.14	0.12	0.0004**	3.57	0.21 [0.01, 0.03]	
2	Communion	eng	1,536	0.22	0.21	0.2792	1.08	0.06 [0.00, 0.02]	
2	Tentativeness	eng	1,536	−0.02	−0.02	0.1815	1.34	0.08 [0.00, 0.01]	
M	Accuracy (LSA)	swe	2,384	−0.09	0.02	0.0000**	−9.50	−0.45 [−0.13, −0.09]	1.00
M	Accuracy (BERT)	swe	2,384	−0.05	0.01	0.0000**	−5.78	−0.28 [−0.08, −0.04]	1.01
M	Valence	swe	2,369	6.08	6.10	0.5036	−0.67	−0.03 [−0.08, −04]	
M	Dominance	swe	2,369	0.03	0.03	0.5941	−0.53	−0.03 [−0.01, 00]	
M	Abstract	swe	2,369	0.14	0.12	0.0003**	3.65	0.18 [0.01, 0.02]	
M	Communion	eng	2,382	0.22	0.21	0.0056*	2.77	0.13 [0.00, 0.02]	
M	Tentativeness	eng	2,382	−0.02	−0.02	0.1966	1.29	0.06 [0.00, 0.01]	

The rows in the table show semantic measures of accuracy divided into studies. The columns shows the Study [1, 2 or Mega (M)], measures prediction of accuracy based on LSA [accuracy (LSA)] or on BERT [accuracy (BERT)], language that the analysis is conducted in [Swedish (swe) or English (eng)], number of data points (N), mean value of measure for incorrect statements (I) or correct statements (C), probability that the incorrect and correct statements differ (p) based on *t*-test with the *t*-values (*t*), level of significance (* < 0.05, ** < 0.001), Cohen’s *d’* (*d’*), lower [CI(l)] and upper [CI(u)] confidence interval, and mean squared error (MSE).

#### 2.3.2 Semantic representations

We next turned to examine if incorrect and correct statements differed in relation to specific semantic representations (Hypotheses 2–4). As the results for the accuracy predictions were similar for the LSA and BERT models, we limit, for the sake of space limitations, these analyses to the LSA model. We first calculated a semantic similarity score for correct and incorrect statements, respectively. This semantic similarity score was calculated as the dot product between the specific semantic representation vector (communion/positive valence/dominance) and the incorrect and correct semantic vector. We then t-tested the difference of these semantic similarity scores. Results are shown in Table 1. In line with hypotheses, results showed that incorrect statements were significantly more closely related to a *communion* representation, whereas correct statements were significantly more closely related to a *dominance* representation. Results showed no statistically significant result of accuracy on a *positive valence* representation.

Taken together, these results support the hypothesis that the semantic space differs for correct and incorrect memories. We thus replicate the finding by Sarwar et al. (2015) with both a new sample, but more importantly, with natural verbal output following an eyewitness interview, rather than written down statements about an event. We also found that both of our analyses of semantic space (BERT, LSA) were able to predict accuracy with statistically significant effects. Moreover, our results showed an effect size of *d* = 0.37 (LSA), which is more sizable than the correlation coefficient found obtained by Sarwar et al. (2015; *r* = 0.04). A straightforward explanation for these differences is that oral testimonies allow for a greater variation in ways to express oneself compared to writing, which could involve more cues to accuracy.

Regarding the relation between accuracy and specific semantic representations, results were largely in line with hypotheses, as incorrect statements were more closely related to a communion representation, whereas correct statements were more closely related to a dominance representation. This supports the idea of compensating a negative aspect in the stereotype content model by boosting another (Holoien and Fiske, 2013; Lindholm and Yzerbyt, 2018). Effect sizes were small-to-medium (*d = 0*.27 and 0.31), which is not surprising, as (a) testimonies are not a typical situation where someone would boast (i.e., express dominance) and as (b) witnesses are unlikely to express incorrect memories they believe to be incorrect, thus limiting the possible feeling of lacking competence, which in turn limits the need to promote communion. The latter explanation might also give some indication as to why no effect was obtained for positive valence.

## 3 Study 2

In this study, we aimed to replicate the findings in Study 1 with a new sample. That is, we (1) expected correct statements to occupy a different semantic space compared to incorrect statements. Moreover, we expected that incorrect and correct statements would differ semantically in relation to the communion, positive valence and dominance representations. Specifically, we hypothesized (2) that incorrect statements would be more closely related to communion and (3) positive valence, whereas (4) correct statements would be more closely related to dominance. Additionally, we wanted to explore potential linguistic differences between correct and incorrect statements further. We therefore added abstractness as an additional semantic representation. This idea was inspired from reality monitoring theory (Johnson and Raye, 1981), which postulates that people judge memories with greater sensory and temporal details as real, and judge memories with references to cognitive operations as imagined. Parallels can be drawn between correct statements and real memories on one hand, and incorrect statements and imagined memories on the other hand, as correct statements are recollections of that which *did* happen, whereas incorrect statements are recollections of that which *did not* happen. As cognitive operations reflect more higher-order functions (i.e., reflections on the memory itself) we expected (5) incorrect memories to be more closely related to an abstract semantic representation. This study has been preregistered^2^.

### 3.1 Method

#### 3.1.1 Dataset

As the semantic analyses work better with greater datasets, this dataset contains combined data from two studies, both conducted in Swedish. The first half of the dataset comprised witnesses in Study 2 from Lindholm et al. (2018), specifically the participants that had viewed a mock crime of a stabbing attack (*n* = 10; *M*_age_ = 24.90, *SD* = 6.42; 50% men). The second half of the dataset comprised witnesses (*n* = 22; *M*_age_ = 24.50, *SD* = 4.97; 32% men) from Gustafsson et al. (2019), who had viewed the same mock crime video. All interviews went through the same transcription and coding procedure as described in Study 1 (see Lindholm et al., 2018; Gustafsson et al., 2019 for full descriptions of the procedures). This combined dataset yielded a total of 1,541 statements (75.47% correct).

The study was conducted in full in accordance with the ethical principles outlined in ALLEA (All European Academies) (2017), the ethical principles outlined on http://www.codex.vr.se/, and with the 1964 Helsinki declaration and its later amendments. The studies did not include factors that require ethical vetting according to Swedish legislation on research ethics Svensk författningssamling (2003). All participants had given informed consent to participate.

#### 3.1.2 Data analysis

Data analyses were identical to those carried out in Study 1, with the addition of an analysis for the abstractness semantic representation. This abstractness representation was created with the same procedure as the semantic representations in Study 1, using the Swedish normwordlist (Waldhauser, 2022). Examples of abstract words were “time” and “plan” whereas examples of concrete words were “pizza” and “snake.”

### 3.2 Results and discussion

#### 3.2.1 Accuracy and semantic spaces

To test the first hypothesis that correct and incorrect differed in semantic space, we again used t-tests for the BERT and LSA-models. The results were statistically significant both with the BERT analysis, *t*(1534) = −3.52, *p* < 0.001, *d* = 0.21, *MSE* = 1.07 (see Table 1), and with the LSA analysis, *t*(1534) = −8.29, *p* < 0.001, *d* = 0.48, *MSE* = 1.06 (see Table 1).

#### 3.2.2 Semantic representations

As in Study 1, we investigated if correct and incorrect statements differed in relation to specific semantic representations (Hypotheses 2–5). Again, we used t-tests to examine the semantic scores for correct and incorrect statements in relation to each representation. Results are shown in Table 1. In line with hypotheses, the results showed that incorrect statements were more closely related to an abstractness dimension. Contrary to hypotheses, results showed no significant results for *communion, positive valence,* nor *dominance.*

Overall, we replicated the results of the first study, demonstrating that correct and incorrect statements differ in semantic content. This was shown both with the BERT analysis and the LSA analysis. However, regarding semantic representations and accuracy, we did not replicate the findings from Study 1 in which incorrect statements were more closely related to communion and correct statements more closely related to dominance (see Table 1). Instead we found that abstractness was higher for incorrect statements. This latter finding corroborates findings on reality monitoring (Hashtroudi et al., 1990; Stromwall and Granhag, 2005; Kensinger and Schacter, 2006; Sporer and Sharman, 2006), in which incorrect statements are uttered with greater “cognitive operations” than correct memories, which should entail more abstract ideas.

## 4 Mega analysis

Next, we decided to pool the data from Study 1 and 2 and make a mega analysis (Eisenhauer, 2021) out of both datasets (*N* = 2,342, accuracy rate = 75.79% correct). Such an analysis should give a more representative picture of the relation between semantic space and eyewitness accuracy due to the larger sample. Furthermore, the larger dataset should allow for better predictions, as the machine-learning algorithm (as specified in Kjell et al., 2019) can learn from more data.

We conducted the same tests as in the first two studies, that is, examined the semantic spaces of correct and incorrect statements with BERT and LSA, as well as examined potential differences in specific semantic representations (communion, dominance, positive valence, abstractness). Additionally, we decided to test if correct and incorrect statements differed in relation to a *tentativeness* semantic representation. The idea for this is straightforward: in the studies from which these datasets originated (Lindholm et al., 2018; Gustafsson et al., 2019), results showed that incorrect statements were expressed with more indications of effort (“effort cues”) than correct statements. One of these types of effort cues was *hedges*, that is, hesitations and commitment avoidance such as “I think,” “perhaps.” These results were based on effort cues that had been manually coded. In this study, we wanted to examine if these results could also be obtained without manual coding, that is, by data-driven analyses. As a proxy for effort/hedging, we used the “tentativeness” word list from the LIWC dictionary (Pennebaker et al., 2001), and applied the same method for measuring this as for the “communion” word list. This list contains words such as “guess,” “possibly” and “hesitate,” but also “darken” and “mysterious.”

To examine differences in semantic space, we again calculated t-tests with the BERT and LSA-models. The results were statistically significant both with the BERT analysis, *t*(2383) = −5.78, *p* < 0.001, *d* = 0.28, *MSE* = 1.01 (see Table 1), and with the LSA analysis, *t*(2383) = −9.50, *p* < 0.001, *d* = 0.45, *MSE* = 1.00 (see Table 1).

We then tested differences in semantic representations between correct and incorrect statements. Results are shown in Table 1, and showed that incorrect statements were significantly more closely related to the *communion* representation, and the *abstract* representation. The remaining representations were not statistically significant, including the newly tested *tentativeness* representation.

### 4.1 *Post-hoc* analyses

Given that we discovered new potential semantic representations as the research progressed, we decided to examine these predictors *post-hoc* also for the individual data sets. We first examined the data in Study 1, and explored whether incorrect and correct statements differed in *abstractness*. Results showed no significant results (see Table 1). We next examined the *tentativeness* representation, and again found no significant results. Moving on to Study 2, a post-hoc exploratory examination also showed no significant effect of *tentativeness.*

### 4.2 Keyword analysis

**Figure 1:**
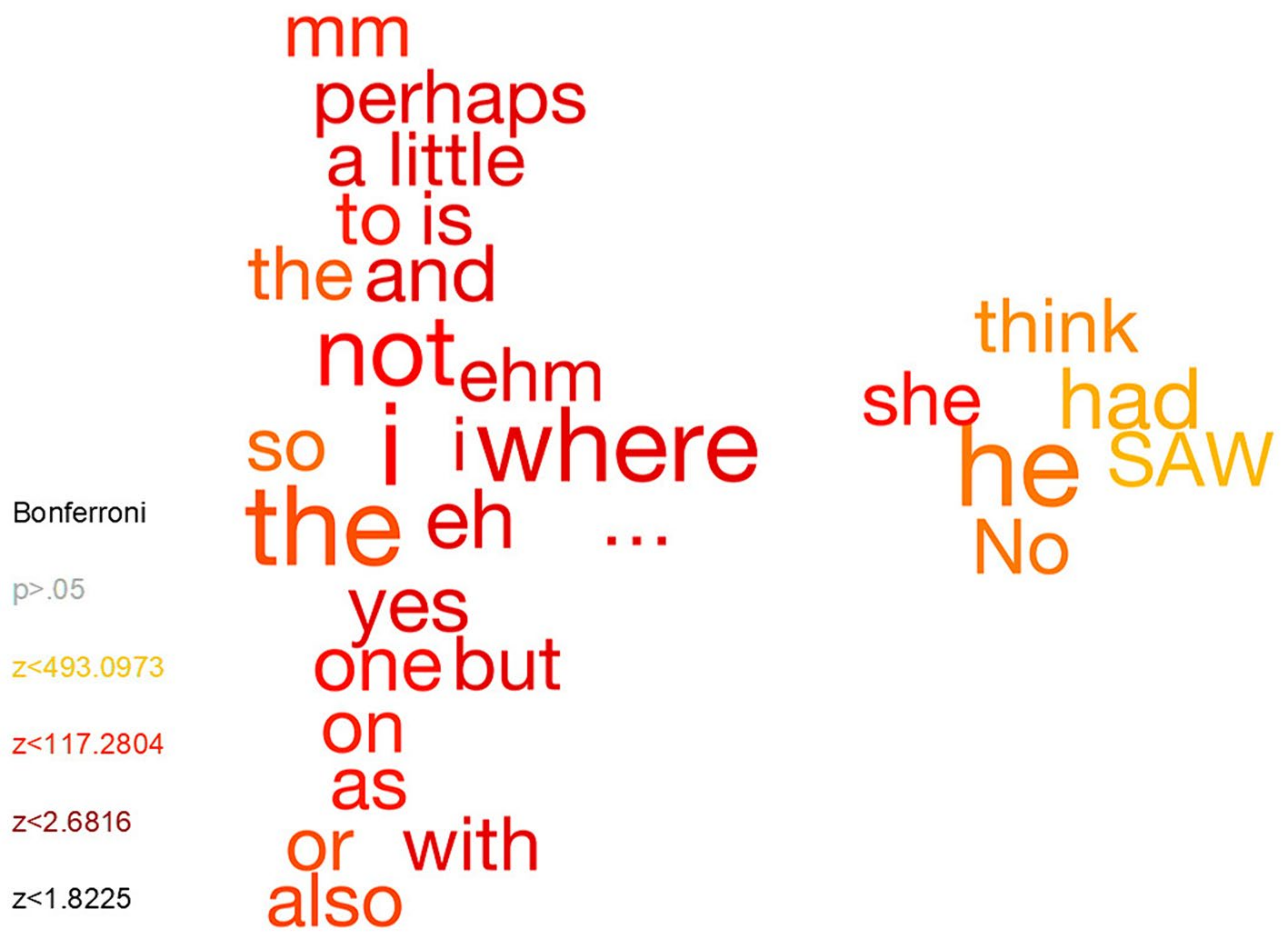
Word cloud analysis of words that are significantly more frequent in incorrect statements **(left side)** and correct statements **(right side)**.

Finally, we wanted to explore if we could single out specific words that discriminate between correct and incorrect statements. This was done by analyzing combined datasets (i.e., the data in the mega analysis) using Chi-square tests testing whether the frequency of each unique word was more common in the correct statements (plotted to the right) compared to incorrect statements (plotted to the left; Figure 1). Words that were significantly different following Bonferroni correction for multiple comparisons were included. The analysis showed that incorrect statements were uttered more commonly with high frequency short function words (e.g., “a,” “to,” “the”), words related to uncertainty, ambiguity, or doubt (e.g., “perhaps,” “also,” “mm,” “eh,” “ehm”) and pronoun coding for the participants (“I”). Correct statements were instead uttered more commonly with pronouns related to third persons (“he” and “she”).

## 5 General discussion

In these studies, we examined if correct and incorrect statements in verbal testimonies differed in linguistic content. Results from both Study 1 and Study 2 supported this idea, as did results from the mega analysis, suggesting that accuracy in eyewitness testimony can be predicted with semantic content. We also examined the relationship between accuracy and semantic representations with the goal to find potential explanations in the type of semantic content that separate correct and incorrect statements (see Table 1). Here results were rather inconsistent across studies, but the mega analysis showed that incorrect statements contained verbal content that was more closely related to semantic representations of communion and abstractness, partly supporting previous studies (e.g., Hashtroudi et al., 1990). We discuss these findings below.

### 5.1 Predicting accuracy with BERT and LSA

In these studies, we attempted to predict accuracy by creating and analyzing semantic spaces wherein correct and incorrect statements can be located. We utilized two methods to do so, BERT and LSA. This double analysis allowed us to get a greater glimpse into the reliability of the findings, as the two methods operate with slightly different methods (for details, see Landauer, 2007; Devlin et al., 2018). Overall, results appeared fairly similar with both methods, with small-to-medium effect sizes (see Table 1).

The relationship between semantic space and accuracy in testimonies have (to the best of our knowledge) only been investigated once before, by Sarwar et al. (2015). Whereas they used written testimonies, we analyzed transcribed verbal testimonies. Our results were overall in line with the findings by Sarwar et al. (2015), that is, that correct and incorrect memories occupy different semantic representations. Moreover, our obtained effect sizes (around *d* = 0.30) appears more sizable than the correlation coefficient found obtained by Sarwar et al. (2015; *r* = 0.04), suggesting that oral testimonies are preferable to written testimonies when predicting accuracy from linguistic content. Taken together, these studies suggest that semantic analyses using n-grams can be fruitful for predicting accuracy in eyewitness testimony and thus adds a new avenue of use in forensic psychology, in addition to the previous use in determining authorship of written texts (e.g., Johnson and Wright, 2014; Nini, 2018).

### 5.2 Examining proximity to specific semantic representations

In addition to predicting accuracy with semantic representation, we attempted to identify what this semantic representation could consist of. To do so, we first selected and created specific semantic representations relating to aspects that we expected to be more common in either correct or incorrect statements, and then examined if correct and incorrect statements differed in their proximity to these representations. Although we found several statistically significant effects, they did not replicate across studies (see Table 1). To get the best representation of these data, we also examined these semantic representations in the pooled mega analysis. In this final analysis, we found that incorrect statements were more closely related to semantic representations consisting of communion and abstractness, in accordance with predictions. Surprisingly, we did not obtain any noticeable effects for the other representations (i.e., valence, dominance, tentativeness). One explanation is that these semantic representations do not map well to the expressions used when recalling witnessed events. This idea is somewhat supported by the fairly low dot-product values (i.e., means for correct and incorrect statements; see Table 1).

A motivation for this study was to examine if automatic computational analyses could achieve results comparable to the costlier manual coding. In the mega analysis, we examined the proximity of accuracy to a “tentativeness” representation, but did not find that it was more closely related to incorrect statements, as expected. The same nonsignificant result was found when post-hoc testing this also for Study 1 and 2 separately. Thus, the findings do not match original analyses of these data (Lindholm et al., 2018; Gustafsson et al., 2019), in which coders manually coded expressions of effort, including *hedges* (“I think,” “maybe”), which they found to be more common in incorrect statements. Thus, it appears that there is a value to manual coding that is not captured in the automatic computational analysis. However, a reservation to this conclusion is that the tentativeness representation was examined in English, using Google translate to convert the testimony statements to English. A fruitful endeavor for future studies is thus to examine English-speaking witnesses.

### 5.3 Word cloud

A word cloud was conducted to examine individual words that were more common in incorrect and correct statements, respectively. Interpreting the results available in Figure 1 suggest that certain words pertaining to hedging (“perhaps”), delays (“…”) and non-filler words (“uh,” “uhm”) were more common in incorrect statements, which corroborate the previous coding of these data (Lindholm et al., 2018; Gustafsson et al., 2019). However, the hedge “think” surprisingly appears to be more common in correct responses. It is also possible that the more common “I” in incorrect statements reflect a self-refence to cognitive operations (“I think I was imagining”/“I never had time to…”), which would be in line with reality monitoring (see Hashtroudi et al., 1990; Stromwall and Granhag, 2005; Kensinger and Schacter, 2006; Sporer and Sharman, 2006). A further interpretation in line with reality-monitoring is the word “saw” (past tense of “see,” not the tool) showing up more often in correct statements, which could suggest more sensory details in correct statements. There also appear to be more conjunction words in incorrect statements (“and,” “or,” “but”), potentially indicating that longer sentences are more likely to be incorrect, perhaps due to containing more (fine-grained) details.

### 5.4 Practical implications

Our study has found evidence that the semantic representation of incorrect statements differs from correct statements. However, a reasonable question is to what extent this is practically useful, as the differences are rather small. We want to stress that—given the current data—the suggested algorithm should not be decisive in discriminating between incorrect or correct statements in important real-life settings, such as court decisions. However, we believe that the proposed methods may provide additional information that might otherwise be overlooked when researching accuracy. For example, the current results suggest that we could potentially get a more complete picture of accuracy by examining the type of information in a statement (e.g., communion, abstractness and tentativeness).

### 5.5 Limitations

Contexts that are different from the ones studied here, or different levels of difficulty of remembering the asked for information, may limit the generalizability of our findings. Furthermore, there are limitations related to ecological validity, where the data used to obtain the testimonies in this study were carried out in a lab environment, in which participants watched a staged crime and were able to keep their full attention on the event. This is not representative of many real-life witnessed events (see Garrett, 2011) and these results can therefore not be directly generalized to real-life eyewitnesses. A clear avenue for future research is to examine the semantic content of statements from real eyewitnesses.

A final limitation is the limited sample used in these studies. Although the total sample in the mega analysis comprised a sample of 2,342 statements, these came from only 66 participants. To establish better reliability of findings, more analyses of witness testimonies are needed.

## 6 Conclusion

Overall, our results indicate that incorrect memories are verbalized with words that differ semantically from those uttered when recalling correct memories. This difference in semantic content could be related to communion, abstractness and, which incorrect statements were more closely related to. However, limited replications warrant any strong conclusions. Nonetheless, given the critical role of eyewitness testimony in the legal context, and the generally low ability of fact-finders to estimate the accuracy of witness statements (e.g., Lindholm, 2008; Gustafsson et al., 2021), our results strongly call for further research on semantic differences in the content of correct and incorrect testimonies.

## Data availability statement

The datasets presented in this study can be found in online repositories. The names of the repository/repositories and accession number(s) can be found in the article/supplementary material.

## Ethics statement

Ethical approval was not required for the studies involving humans because the study was conducted in full in accordance with the ethical principles outlined in ALLEA (All European Academies) (2017), the ethical principles outlined on http://www.codex.vr.se/, and with the 1964 Helsinki declaration and its later amendments. The studies did not include factors that require ethical vetting according to Swedish legislation on research ethics Svensk författningssamling (2003). All participants had given informed consent to participate. The studies were conducted in accordance with the local legislation and institutional requirements. The participants provided their written informed consent to participate in this study.

## Author contributions

PUG: Methodology, Data curation, Writing – original draft, Writing – review & editing, Visualization, Project administration, and Supervision. SS: Conceptualization, Methodology, Software, Formal analysis, Writing – review & editing, Visualization. TL: Conceptualization, Methodology, Investigation, Resources, Writing – review & editing, Supervision.

## Footnotes

^1^ https://huggingface.co/docs/transformers/v4.36.1/en/multilingual#bert

^2^ https://osf.io/ku659
